# Efficient derivation of knock-out and knock-in rats using embryos obtained by *in vitro* fertilization

**DOI:** 10.1038/s41598-019-47964-1

**Published:** 2019-08-09

**Authors:** Arata Honda, Ryoma Tachibana, Kazuya Hamada, Kohtaro Morita, Naoaki Mizuno, Kento Morita, Masahide Asano

**Affiliations:** 1 Institute of Laboratory Animals, Kyoto University Graduate School of Medicine, Yoshidakonoe-cho, Sakyo-ku, Kyoto 606-8501 Japan; 2 RIKEN BioResource Research Center, Tsukuba, Ibaraki 305-0074 Japan; 3 Division of Stem Cell Therapy, Institute of Medical Science, University of Tokyo, Minato-ku, Tokyo 108-8639 Japan

**Keywords:** Rat, Genetic engineering, Development

## Abstract

Rats are effective model animals and have contributed to the development of human medicine and basic research. However, the application of reproductive engineering techniques to rats is not as advanced compared with mice, and genome editing in rats has not been achieved using embryos obtained by *in vitro* fertilization (IVF). In this study, we conducted superovulation, IVF, and knock out and knock in using IVF rat embryos. We found that superovulation effectively occurred in the synchronized oestrus cycle and with anti-inhibin antiserum treatment in immature rats, including the Brown Norway rat, which is a very difficult rat strain to superovulate. Next, we collected superovulated oocytes under anaesthesia, and offspring derived from IVF embryos were obtained from all of the rat strains that we examined. When the *tyrosinase* gene was targeted by electroporation in these embryos, both alleles were disrupted with 100% efficiency. Furthermore, we conducted long DNA fragment knock in using adeno-associated virus and found that the knock-in litter was obtained with high efficiency (33.3–47.4%). Thus, in this study, we developed methods to allow the simple and efficient production of model rats.

## Introduction

The brown rat (*Rattus norvegicus*) was first used as a laboratory animal in Europe during the 1850s and it was the first mammalian species to be domesticated for scientific purposes^1^. The size and simple handling of rats are major characteristics that may have motivated the selection of rats for early animal experiments in studies of breeding, behaviour, psychology, nutrition, endocrinology, genetics, and other research. Moreover, comprehensive database platforms related to laboratory rats have been developed, such as the National BioResource Project-Rat in Japan and Rat Genome Database in the USA^1–3^. Rats have contributed greatly to the development of therapeutic agents and regimens as human disease models, but their use in gene modification research is not as advanced as that of mice because of difficulties conducting fundamental developmental engineering techniques such as superovulation, *in vitro* fertilization (IVF), and pluripotent stem cell manipulation. Superovulation is one of the most important techniques for the effective production of genetically modified rats. However, only an average of 2.2 oocytes per rat can be recovered using superovulation methods, even in the valuable inbred Brown Norway (BN) strain, where over 90% of its genome sequence has been determined^2,4^. Mouse reproduction techniques such as IVF and embryo manipulation were established in the 1970s, while the development of gene knock-out (KO) mice using embryonic stem cells occurred before 1990; both are critical tools for understanding gene functions^5–7^. In addition, the first generation of gene KO rats were obtained via homologous recombination in 2010, which was almost 20 years after the production of KO mice^8^. Recently developed synthetic sequence-specific nucleases (gene editors), such as zinc finger nucleases, transcription activator-like effector nucleases, the clustered regularly interspaced short palindromic repeat (CRISPR/Cas) system, and engineered mega-nucleases have all been used to genetically modify the rat genome^9–12^. Several studies have reported the successful production of rat offspring by IVF^13–17^, but all of the rats with genomic modifications produced via micromanipulation, electroporation, and the genome editing via oviductal nucleic acids delivery system (GONAD) were derived by *in vivo* fertilization (mating) rather than IVF^9–12,18–21^, which reflects the difficulty of efficiently producing rats using IVF.

In this study, we successfully developed methods for the efficient and simple (free of micromanipulation) production of KO and knock-in (KI) rats using superovulation and for obtaining IVF-derived oocytes in several rat strains, including BN. These methods facilitate the production of genetically modified rats by researchers who require model rats.

## Results

### Oestrus cycle synchronization and inhibin suppression enhanced superovulation in several rat strains

**Figure 1:**
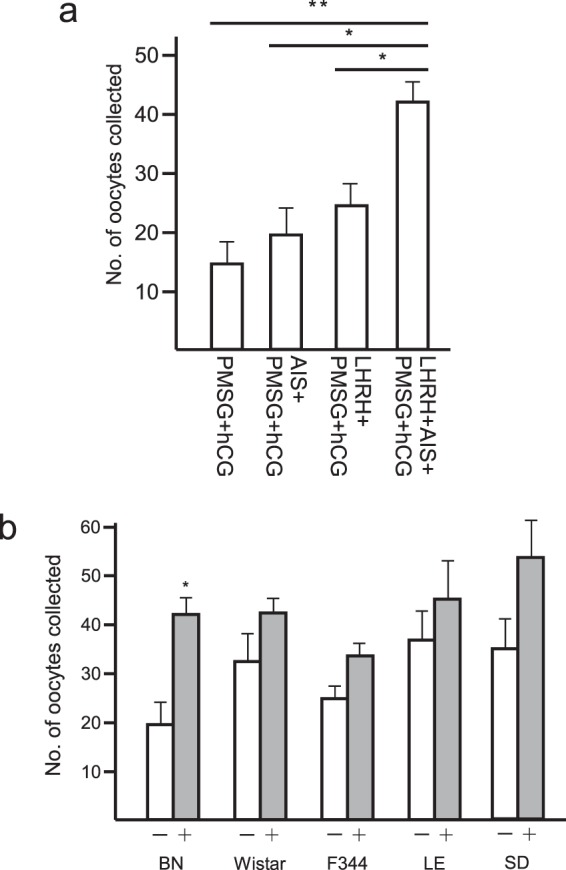
(**a**) Changes in the number of oocytes collected from oviducts of BN females that superovulated after the injection of LHRH, AIS, PMSG, and hCG. Values are the mean ± standard deviation. The PMSG- and hCG-treated groups differed significantly to the other three groups (*p < 0.05, **p < 0.01). (**b**) Effects of LHRH on superovulation by various rat strains. The numbers of oocytes collected from the oviducts of BN, Wistar, F344, LE, and SD strains in the absence (−) and the presence (+) of LHRH are shown. PMSG, AIS, and hCG were administrated irrespective to the LHRH treatment. Values are the mean ± standard deviation. The LHRH-treated group differed significantly from that without LHRH (*p < 0.05).

Superovulation techniques must be established for a wide range of rat strains in order to develop effective reproduction techniques. In previous studies, superovulation was generally induced in rats using pregnant mare serum gonadotropin (PMSG) and human chorionic gonadotropin (hCG)^22^. However, it is well-known that the usual superovulation treatment is not effective in some strains. Taketsuru *et al*. reported that for BN—which is a representative strain and its genomic sequence has been determined as that for the rat—only 2.2 ± 1.4 oocytes could be obtained from sexually mature females (8–13 weeks of age) using the conventional treatment (PMSG–hCG injection)^4^. To overcome this problem, we evaluated the previously reported treatments that are known to accelerate superovulation, *i*.*e*., oestrus cycle synchronization (administration of luteinizing hormone-releasing hormone (LHRH)) and passive immunoneutralization of endogenous inhibin (administration of anti-inhibin serum (AIS)), in immature female rodents^23–28^. When superovulation was induced by injecting PSMG-hCG into immature BN rats, 14.3 ± 3.9 oocytes were collected (Fig. 1a and Table 1), which suggests that the immature BN rats ovulated more effectively than the adult BN rats^4^. When AIS was applied simultaneously with PMSG and hCG (AIS/PMSG–hCG) in immature BN rats, 19.9 ± 4.5 oocytes were collected. After oestrus cycle synchronization was induced by LHRH treatment and the injection of both PMSG and hCG (LHRH-PMSG-hCG), 22.3 ± 3.7 oocytes were collected. Moreover, superovulation after synchronizing the oestrous cycle with LHRH and inhibin suppression (LHRH-AIS/PMSG-hCG) allowed us to collect 42.0 ± 3.7 oocytes from immature BN rats. No significant differences were detected, but oestrus cycle synchronization had positive effects on superovulation in the Wistar, F344, Long-Evans (LE), and Sprague Dawley (SD) strains (Fig. 1b and Table 2). Thus, treatment with LHRH and AIS to stimulate superovulation was employed in our subsequent experiments.

**Table 1:** Effects of LHRH and AIS on superovulation by BN rats.

	Group	BN
No. of oocytes collected	PMSG-hCG	14.3 ± 3.9 (n = 6)
	AIS-PMSG-hCG	19.9 ± 4.5 (n = 9)
	LHRH-PMSG-hCG	22.3 ± 3.7 (n = 6)
	LHRH-AIS-PMSG-hCG	42.0 ± 3.7 (n = 9)

Values are mean ± S.D.

**Table 2:** Effect of LHRH on the superovulation by various rat strains.

	Group	BN	Wistar	F344	LE	SD
No. of oocytes collected	LHRH (−)	19.9 ± 4.5 (n = 9)	32.6 ± 6.5 (n = 8)	25.0 ± 3.2 (n = 6)	36.6 ± 7.3 (n = 5)	35.3 ± 6.0 (n = 6)
	LHRH (+)	42.0 ± 3.7* (n = 9)	42.5 ± 2.5 (n = 8)	33.3 ± 3.8 (n = 11)	45.5 ± 8.3 (n = 6)	53.9 ± 6.1 (n = 7)

Values are mean ± S.D. *P < 0.05.

### IVF using superovulated oocytes

To develop an efficient reproduction technique in rats, we confirmed the developmental potential of the superovulated oocytes based on their application in IVF. Several studies have shown that the cumulus–oocyte complexes (COCs) need to be collected immediately after sacrificing female rats^13,15^. Thus, the oviductal ampullae were removed under anaesthesia to avoid prolonged manipulation after sacrifice. To evaluate the effect on the IVF rate of reduced glutathione (GSH), which is an enhancer of fertilization by frozen–thawed sperm^29,30^, the COCs were placed in a drop of human tubular fluid (HTF) medium with or without GSH and then inseminated with pre-incubated sperm (Table 3). The IVF rate was evaluated based on the whole stained mount and it varied in the range of 55.2–98.3% among the rat strains examined. Pre-treatment of the COCs with GSH appeared to enhance the IVF rate in the BN, Wistar, F344, LE, and SD strains. One day after IVF, one- to two-cell embryos were transferred into the oviducts of pseudo-pregnant females. The number of offspring derived from IVF was increased by GSH treatment in BN and F344, but it did not have a positive effect on the Wistar, LE, and SD strains (Table 3).

**Table 3:** Developmental capacity of IVF-derived embryos in various rat strains.

Strain	GSH treatment	No. of fertilized oocytes/examined (%)	No. of embryos transferred	No. of offspring (%)
BN	(−)	62/83 (74.7)	49	12 (24.5)
	(+)	49/57 (86.0)	192	54 (28.1)
Wistar	(−)	65/77 (84.4)	63	18 (28.6)
	(+)	74/79 (93.7)	90	19 (21.1)
F344	(−)	32/58 (55.2)	85	14 (16.5)
	(+)	36/52 (69.2)	90	23 (25.6)
LE	(−)	59/60 (98.3)	56	12 (21.4)
	(+)	62/71 (87.3)	50	6 (12.0)
SD	(−)	54/63 (85.7)	52	11 (21.1)
	(+)	56/60 (93.3)	52	10 (19.2)

### Efficient generation of KO rats by electroporation of CRISPR/Cas9 in IVF embryos

**Figure 2:**
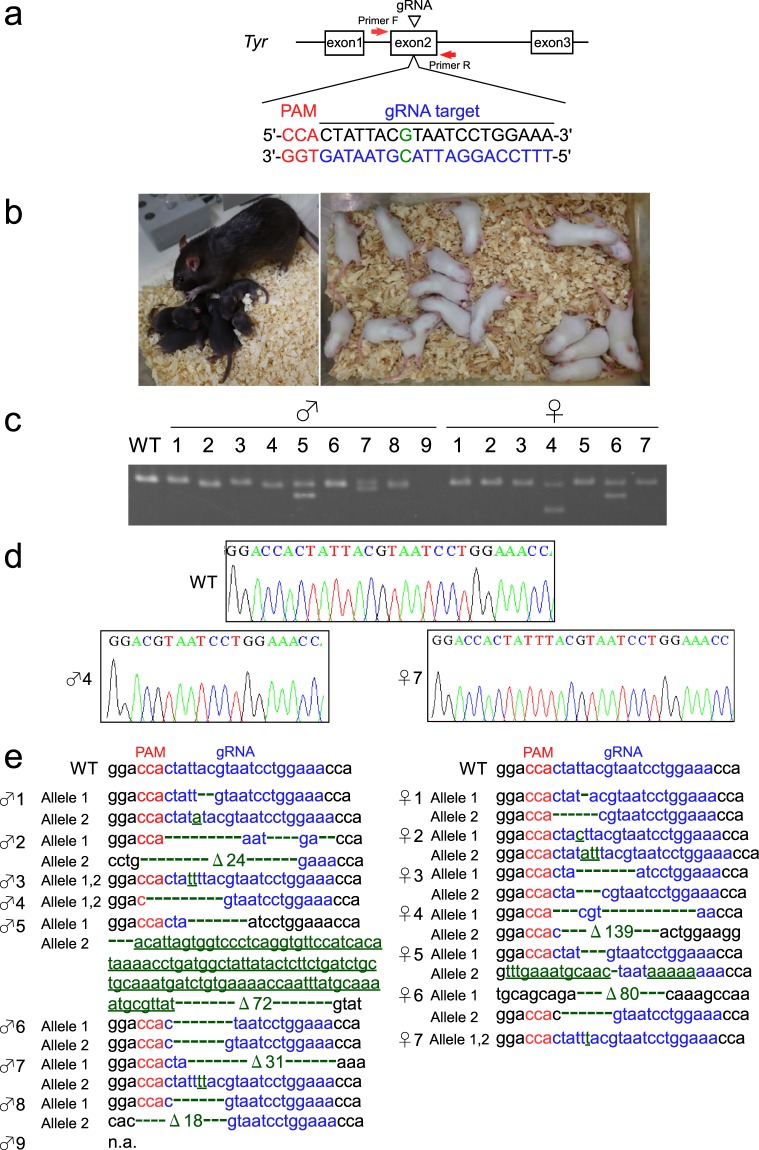
(**a**) Schematic representation of the *Tyr* locus. The target sequence of *Tyr* exon 2 recognized by gRNA is overlined and the protospacer adjacent motif (PAM) sequence is marked in red. The target nucleotide ‘G’ marked in green is a key nucleotide for tyrosinase activity, and nucleotide replacement at this position often leads to an albino phenotype. PCR primers used to evaluate the indel mutations around the target site are indicated as red arrows. (**b**) BN offspring obtained after electroporation of CRISPR/Cas9 complex in IVF embryos. By contrast with the normal (dark brown) BN rats (left panel), all of the offspring obtained had the albino phenotype (right panel). (**c**) PCR products amplified from genomic DNA of offspring with the albino phenotype represented in (**b**) showed a wide variety of band patterns. (**d**) Representative Sanger sequencing at the gRNA target site of male-4 (♂ 4) and female-7 (♀ 7). (**e**) Sequence analysis of PCR products amplified from the genomic DNA of offspring with the albino phenotype represented in (**b**). All of the sequences examined contained a wide variety of biallelic indel mutations (green). Insertions are underlined. n.a.: not amplified due to problems with PCR.

To evaluate the feasibility of genome editing using IVF rat oocytes, the rat coat colour *tyrosinase* gene (*Tyr*) was disrupted via the delivery of the CRISPR/Cas9 complex by electroporation into GSH-treated BN oocytes. We used gRNA^18,21,31^, which achieves a targeted mutation of 896G in exon 2 of the *Tyr* gene, and which has also been reported in human oculocutaneous albinism type 1A with a lack of pigmentation^32^. The birth rate was 28.7% (49/171); thus, introducing the CRISPR/Cas9 complex by electroporation made no significant difference to the IVF embryos without electroporation (28.1%: 54/192) (Tables 3, 4 and S2). Surprisingly, all of the pups (49/49) obtained had the albino phenotype and 46 of the pups had biallelic mutations around the targeted site (Figs 2b–e, S2 and Table 4). None of the wild type alleles were found in any of the pups that we sequenced. The genomic sequence could not be determined for the remaining three albino pups (3/49) due to problems with PCR amplification around the targeted sequence. In addition, no off-target mutations were detected away from the target site that had previously been indicated^31^ in any of the pups examined by sequencing of the candidate off-target site. These results suggest that IVF embryos are effective for the generation of gene KO rats.

**Table 4:** Knock-out efficiency of rat offspring after zygote genome editing using electroporation.

Target gene	No. of embryos electroporated	No. of embryos transferred	No. of offspring (%)	No. of KO offspring with albino (%)
*Tyr*	224	171	49 (28.7)	49 (100.0)

This high genome editing efficiency in IVF embryos could be explained by the long period for which the CRISPR/Cas9 complex was applied to the target genome sequence in a suitable developmental stage after fertilization. The IVF embryos were subjected to electroporation at 8–10 h after insemination, and thus the CRISPR/Cas9 complex may have been active for a long time in the G1–M phase, where the joining of non-homologous ends probably occurred. To confirm correlation to the genome editing efficiency and the timing for CRISPR/Cas9 delivery, the CRISPR/Cas9 complex was introduced to the IVF embryos 10 h, 14 h, and 18 h after insemination. Surprisingly, all pups obtained showed an albino phenotype regardless of the timing of electroporation of CRISPR/Cas9 (Table S2 and Fig. S2). These results suggested that high genome editing efficiency in IVF embryos is not influenced by the period (8–18 h) of exposure of the CRISPR/Cas9 complex to the target genome sequences.

### Manipulation-free generation of large-fragment KI rats via adeno-associated virus (AAV) transfection in IVF embryos

**Figure 3:**
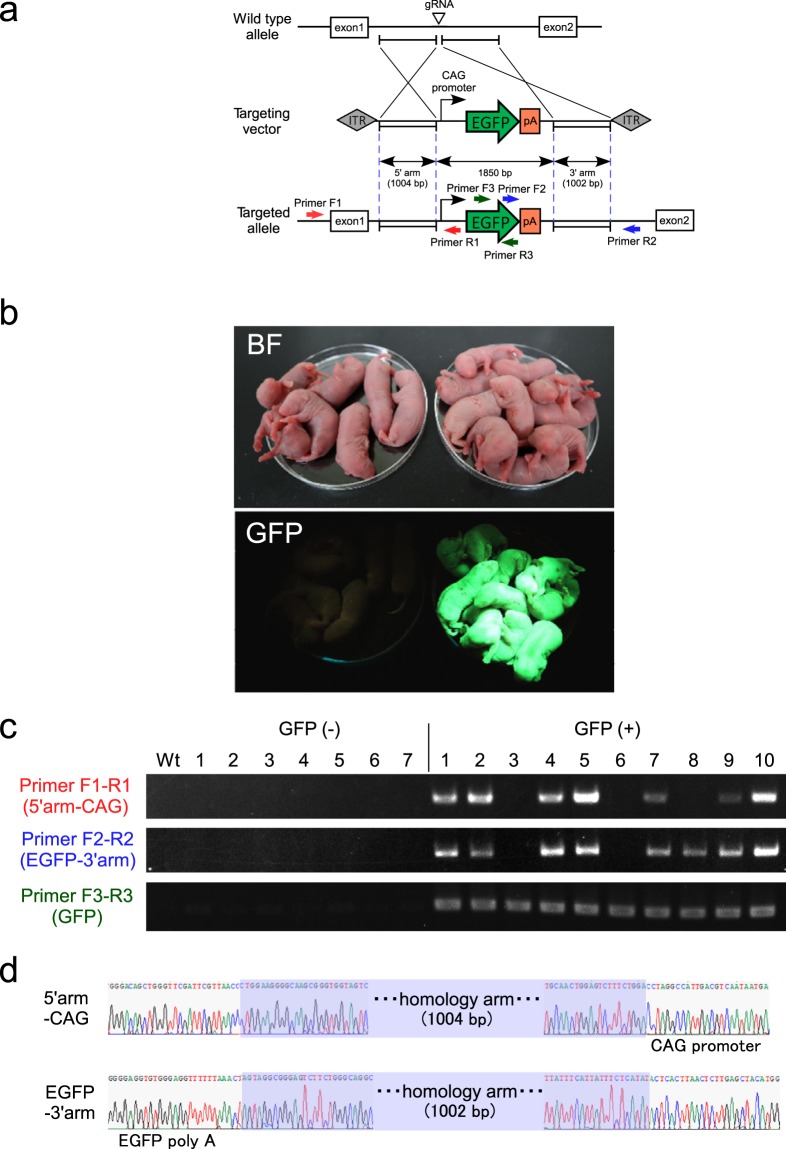
(**a**) Schematic representation of the targeting strategy. The donor ssAAV vector containing the 1,850-bp CAG-EGFP cassette was flanked by approximately 1,000-bp homology arms next to the gRNA target. Arrows indicate the primers used for genomic analysis. (**b**) Fluorescence observation of neonates without (left) or with (right) GFP signals. (**c**) Representative genotyping of neonates without (GFP (−): 1–7) and with (GFP (+): 1–10) GFP signals. Primers flanking the 5′-(Primer F1-R1) and 3′- (Primer F2-R2) junctional regions, and GFP cassette (Primer F3-R3) were amplified. GFP (+) 1, 2, 4, 5, 7, 9, and 10 had targeted alleles at a precise locus. GFP (+) 8 was a putative partial insertion. GFP (+) 3 and 6 had possible random integrations of the targeting vector elsewhere in the genome. (**d**) Representative Sanger sequencing of the precise insertion of knock-in cassettes in an offspring, GFP (+)-1. Homology direct repair was confirmed by Sanger sequencing of the 5′ junctional region (upper sequence) and 3′ junctional region (lower sequence). Blue box, homology arm.

The targeted knock in of DNA fragments in rats has been achieved successfully and effectively by microinjection, GONAD, and AAV transfection^18,21,31,33,34^. However, all of these previously reported KI rats were derived from embryos obtained by *in vivo* fertilization. In this study, we evaluated the method based on AAV transfection in order to establish an efficient and simple strategy for generating large exogenous fragment KI offspring^33^. The production of KI rats via AAV transfection can implement large DNA fragment knock in without advanced training and expensive micromanipulation equipment. In this study, we disrupted the *Rosa*26 site in IVF embryos using gRNA via electroporation and the embryos were then co-incubated overnight with the recombinant ssAAV6 (AAV6_rRosa26_CAG_EGFP) at various virus concentrations (1 × 10^4^, 1 × 10^5^, and 1 × 10^6^ IU/mL) (Table S3 and Fig. S3). In addition, the KI without virus infection but with the electroporation of long double-strand DNA (ldsDNA) was also examined. The next day, embryos at the one- or two-cell stage were cultured by mR1ECM to develop them into morula or blastocysts. No GFP signal or KI allele emerged in the embryos examined after electroporation of ldsDNA and virus infection at 10^4^ IU/mL (Table S3 and Fig. S3b,c). By contrast, GFP signal and KI allele could be detected after transfection at 10^5^ IU/mL (GFP: 62.5%, KI: 25.0%) and 10^6^ IU/mL (GFP: 85.7%, KI: 71.4%). Therefore, one- or two-cell stage embryos which had been transfected with ssAAV6 (10^5^ IU/mL) were then transplanted into pseudo-recipients. Newborn pups were observed under a fluorescent device (Table 5 and Fig. 3b). Precise knock in was achieved in 9/19 pups (47.4%), which exhibited uniform GFP signals over their whole bodies. Two of the pups (2/9) were stillbirths, but they had the exact targeted allele (Fig. S4). Among the GFP-positive pups, 1/19 (5.3%) had a partial insertion and 2/19 (10.5%) exhibited random integration (Table 5 and Fig. 3c). The sequence of knock-in pups (9/19) was analysed to determine the occurrence of precise homology direct repair at the target site (Fig. 3d). All of the GFP-negative offspring (7/7) had the biallelic mutation around the gRNA target site (Fig. S5).

**Table 5:** Knock-in efficiency of rat offspring after zygote genome editing using electroporation followed by AAV transduction.

Target gene	No. of embryos electroporated	No. of embryos cultured with AAV	No. of embryos survived (%)	No. of embryos transferred	No. of offspring (%)	No. of KO offspring without GFP signal (%)	No. of offspring with GFP signal (%)	No. of offspring with partial insertion (%)	No. of offspring with random integration (%)
*Rosa 26*	376	366	349 (95.4)	180	19 (10.6)	7 (36.8)	12 (63.2)	9 (47.4)	2 (10.5)

Next, we examined whether the albino phenotype in Wistar rats can be rescued through IVF-based KI using recombinant ssAAV6 (AAV6_*rTyr*-repair) (Tables S4, S5 and Fig. S6). When the KI efficiency was evaluated on the preimplantation embryos, no KI allele was detected by PCR-restriction fragment length polymorphism (PCR-RFLP) at a virus concentration of 10^5^ IU/mL (Table S4, Figs S6b and S8). Because 25% (4/16) of preimplantation embryos had a KI allele, we transferred one- or two-cell stage embryos that had infected ssAAV6 (AAV6_*rTyr*-repair) at 10^6^ IU/mL. Pigmented eyes were found in 1/3 (33.3%) of the resulting offspring (Table S5, and Fig. S6c). PCR-RFLP and direct Sanger sequencing of PCR products derived from a pigmented rat showing successful KI of *Tyr* locus (Fig. S6d–f). As confirmed in the KI experiment using *Rosa26*, all of the KI-negative offspring (2/2) had the biallelic mutation around the gRNA target site (Fig. S6e). In addition, no off-target mutations were detected away from a target site in any of the pups examined by sequencing of a candidate off-target site. These results indicated the high genome editing (KO/KI) performance of the IVF-derived embryos.

## Discussion

Rats are useful model animals but the reproductive engineering techniques that can be used on them are much more limited than those for mice. In this study, we obtained four major findings. First, we achieved superovulation in several rat strains, including BN, which usually yields a low number of oocytes. Second, these oocytes are suitable for producing offspring by IVF. Third, KO rats could be obtained efficiently from IVF embryos by electroporation. Finally, the IVF embryos were effective for generating KI rats by AAV transfection. These simple and efficient methods for producing model rats will contribute greatly to their development as human disease models.

BN is one of the most important inbred rat strains and the genome sequence of the rat was obtained from the BN strain^2^. However, the average number of natural mating derived BN offspring is low and superovulation is also difficult in this strain, thereby making it unsuitable for model animal production^4,35^. Indeed, Taketsuru *et al*. reported that it is very difficult to induce superovulation in sexually mature BN rats. However, several studies have demonstrated that efficient superovulation can be achieved using immature females^25,36^. In the mouse, it was confirmed that the number of oocytes can also be increased by synchronizing the oestrus cycle and treating with AIS^24^. In rats, it is known that synchronization can be achieved by administering LHRH rather than progesterone, and even in immature rats, the timing of ovulation can be adjusted using LHRH^37^. In this study, we found that the frequency of ovulation increased when we used immature BN rats. Moreover, the administration of both LHRH and AIS synergistically increased superovulation in BN rats. However, treatment with LHRH had only slight positive effects on ovulation in the Wistar, F344, LE, and SD strains.

Clearly, successful IVF has been achieved in rats in previous studies and this technique is feasible^13–17^. However, despite the large number of reports of successful genome editing, all were achieved using fertilized embryos obtained from natural mating, thereby strongly suggesting that it is difficult to obtain suitable offspring via IVF. Aoto *et al*. stressed the importance of collecting oocytes as soon as possible after euthanasia^13^. Therefore, in this study, we obtained offspring by IVF from the strains where the oocytes were collected under anaesthesia, and we were subsequently able to successfully achieve highly efficient genome editing in IVF embryos obtained in this manner. However, although the fertilization rate was very high, the birth rate was less than 30%, so we recognize that this IVF technique needs to be improved further for the availability of frozen–thawed IVF embryos. Modifying the zona pellucida using GSH is known to be effective in mice but the effects differ among strains of rats. Thus, it may be necessary to identify the optimal conditions for each rat strain in terms of the GSH concentration and processing time.

The elimination of *Tyr* has been used frequently for determining the feasibility of genome editing in many animal species^18,38–40^. In this study, we used the same gRNA employed in previous studies to eliminate the rat *Tyr* gene^18,21,31^, where the genome editing efficiency in these previous studies was 14/34 = 41.2% for microinjection, 35/65 = 53.8% for rGONAD, and 17/29 = 58.6% for i-GONAD. In the present study, when we disrupted the *Tyr* gene in IVF embryos using the same gRNA, 89 of the 89 offspring obtained were albino (=100%), and we confirmed that all of the pups examined (46/46) were biallelically mutated. A high genome editing efficiency using IVF embryos was also demonstrated based on the targeted disruption of the *Rosa26* site and the *Tyr* of Crlj:WI, where knock in led to biallelic indel mutations in all non-KI offspring (Tables 5 and S5, Figs S5 and S6e). Because an appropriate condition for electroporation had been optimized for naturally fecundated zygotes according to previous reports^19,20,41^; therefore, we applied it to the IVF embryos. As a result, this condition was much more effective for IVF embryos than naturally fecundated embryos. In previous studies, genome editing was conducted with embryos obtained by *in vivo* fertilization, so these embryos experienced a developmental time lag after their fertilization in each female. Moreover, the GONAD method has a restriction of oocyte collection because the cumulus cells must be loosened to some extent^21,31^. When fertilized embryos are obtained with IVF, it is possible to specify the fertilization timing for most of the embryos, and to obtain homozygous rat embryos efficiently for blastocyst complementation experiments^42–45^.

Microinjection is the main choice for the knock in of long DNA fragments in rats rather than using short fragments such as single-strand oligonucleotides. However, microinjection requires expensive equipment and advanced techniques to obtain stable results. Other methods such as electroporation including the technique for animal knock out system by electroporation or GONAD may be more difficult to achieve the knock in of long DNA fragments because they do not require micromanipulation^19,21^. However, Mizuno *et al*. demonstrated the knock in of long DNA fragments using AAV6^33^, which is an effective method that does not require micromanipulation. In our study using IVF embryos, we also obtained a high knock-in efficiency of 9/19 (47.4%) on *Rosa26*, 1/3 (33.3%) on *Tyr*, and Mizuno *et al*. reported an efficiency of 3/3 (100%). However, the birth rate of the KI embryos obtained by IVF was not very high (10.6% on *Rosa26*, and 7.1% on *Tyr*). The amount of KI offspring produced with AAV transfection was also low (3/56 = 5.4%) despite the fact that Mizuno *et al*. used embryos obtained by *in vivo* fertilization^33^. This reduction in the birth rate may be attributed to the cytotoxic effect of the viral infection.

In this study, we successfully achieved superovulation, IVF, and highly efficient KO/KI using IVF embryos. The methods developed in this study are valuable according to the principle of the 3Rs (reduction, replacement, and refinement), and they are suitable for the simple and efficient production of rat models.

## Materials and Methods

### Animals

Specific pathogen-free Brown Norway/CrlCrlj (BN) rats and Crlj:Wistar (Crlj:WI) rats were purchased from Charles River Japan (Kanagawa, Japan). Jcl:Wistar (Wistar), F344/Jcl (F344), and Jcl:Sprague Dawley (SD) rats were purchased from CLEA Japan (Tokyo, Japan). The Long–Evans (Iar:Long–Evans) (LE) strain was purchased from the Institute for Animal Reproduction (Ibaraki, Japan). Rats were housed in an environmentally controlled barrier room with a 12 h light cycle (7:00 to 19:00) and 12 h dark cycle at a temperature of 24 ± 2 °C and humidity of 50 ± 10%. All of the animal care procedures performed in this study conformed to the Guidelines for Proper Conduct of Animal Experiment and Related Activities in Academic Research Institutions under the jurisdiction of the Ministry of Education, Culture, Sports, Science and Technology of Japan approved by the Animal Research Committee of Kyoto University.

### Superovulation

The female rats were injected intraperitoneally with PMSG (300 IU/kg) (Aska Animal Health Co. Ltd, Tokyo, Japan) and AIS (100 μL) (Central Research Co. Ltd, Tokyo, Japan), before injecting hCG (300 IU/kg) (Aska Pharmaceutical Co. Ltd, Tokyo, Japan) 48–50 h later. MII oocytes were collected from the oviducts at 16–17 h after hCG injection. The rats subjected to oestrus cycle synchronization were injected intraperitoneally with 0.04 mg (dissolved in 200 μL saline) of [des-Gly10, D-Ala6]-LH-RH ethylamide acetate salt hydrate (Sigma-Aldrich) at 52–53 h before PMSG treatment.

### IVF and embryo transfer

IVF was conducted as described previously^13,15^. Briefly, HTF medium purchased from ARK Resource Co. Ltd (Kumamoto, Japan) was covered with paraffin oil (Nacalai Tesque, Kyoto, Japan) and placed in an incubator for 1 h at 37 °C under 5% CO_2_ in air to allow equilibration. After the medium equilibrated, cauda epididymal sperm were placed in a drop and dispersed for capacitation for 1 h, and then the incubated sperm in the drops were employed for insemination at a density of 1.0–2.0 × 10^5^/100 μL. The oviduct ampullae from superovulated females were isolated under anaesthesia, which was achieved by intraperitoneally injecting a mixture of three anaesthetic agents (0.375 mg/kg medetomidine, 2 mg/kg midazolam, and 2.5 mg/kg butorphanol), and immediately removing the surface liquid and blood using a sterilized filter paper, and then placing the ampullae into paraffin oil in an insemination dish. COCs were pulled from the cut oviduct ampullae with hypodermic 26G gauge needles and placed in the inseminated drops. For the oocytes that received pretreatment with reduced GSH, the COCs were removed in HTF medium supplemented with 1.5 mM GSH and incubated for 15 min^30^. The superovulated oocytes were inseminated with sperm from the same rat strain. After insemination of the oocytes with sperm, they were cultured at 37 °C under 5% CO_2_ in air for 6 h. After incubation, the inseminated oocytes were washed to remove the cumulus cells and sperm, and cultured in HTF medium. To determine the fertilization rate, the inseminated oocytes were fixed with 2.5% glutaraldehyde and stained with 1% aceto-orcein to visualize the pronuclei^46^. The oocytes with a male pronucleus or pronuclei and sperm tail(s) in the cytoplasm were considered to be fertilized irrespective of the number of penetrating sperm. At 18 to 24 h after IVF, the fertilized oocytes (one- or two-cell embryos) were transferred to the oviducts of pseudo-pregnant recipient females. The number of offspring born naturally or by caesarean section was counted after 21 days of gestation.

### Knock out by electroporation of IVF embryos

CRISPR/Cas9-mediated gene knock out was performed using a Super Electroporator NEPA21 (NEPA GENE Co. Ltd, Chiba, Japan) as described previously used for naturally fecundated zygotes but with minor modifications^19,20,47^. At 8 to 18 h after IVF, the pronuclear stage BN embryos were placed in a line on the glass chamber (CUY520P5) between the metal plates, which were filled with Opti-MEM medium (Thermo Fisher Scientific, Waltham, MA, USA) containing 4 μM of Alt-R CRISPR-Cas9 crRNA (listed in Table S1), Alt-R CRISPR-Cas9 tracrRNA duplex, and 1.2 μM of Alt-R Cas9 Nuclease V3 (Integrated DNA Technologies, Inc., LA, USA). The gRNA target was rat *Tyr*, 5′-TTTCCAGGATTACGTAATAG-3′. Poring pulse (voltage: 225 V, pulse interval: 50 ms, number of pulses: 4) was selected and the pulse width was adjusted to 0.5, 1.5, and 2.5 ms^19,20,41^. After electroporation, the embryos were transferred to HTF medium and cultured overnight at 37 °C under 5% CO_2_ in air. The following morning, the fertilized embryos (one- or two-cell stage) were transferred to the oviducts of pseudo-pregnant recipient females. The number of offspring born naturally or by caesarean section was counted after 21 days of gestation.

### Targeting vector construction

For *Tyr*-repair of albino rats, a targeting ssAAV vector plasmid, pssAAV_*rTyr*-repair, was constructed. *Tyr* gene was amplified by PCR using the primers listed in Table S1 (Wistar *Tyr* amplification) using peripheral blood cells of Crlj:WI as a template. *Tyr* homology arms were amplified by PCR using the primers listed in Table S1 (5′-homology arm and 3′-homology arm) using the template as above. These homology arms include the *Tyr*_repair cassette, which has corrected sequences containing an in-frame silent mutation to provide an *Sna*BI site for RFLP analysis. Homology arms were inserted into pUC19 using an In-Fusion HD Cloning Kit. After confirmation of the sequence, homology arms and *rTyr*-repair cassette were excised using restriction endonucleases, *Nhe*I-HF and *Mlu*I-HF (New England Biolabs, Japan, Tokyo, Japan), and then ligated with pAAV_MCS2. AAV6_*rTyr*-repair was conducted as reported previously^33^.

### Knock in using CRISPR/Cas9 electroporation followed by infection with AAV vector

Knock in of IVF embryos via AAV transfection was performed using the methods developed for embryos produced by *in vivo* fertilization^33^. We infected the recombinant ssAAV6 (AAV6_*rRosa26*_CAG_EGFP or AAV6_*rTyr*-repair) as described previously^33^. Superovulated Crlj:WI oocytes were inseminated with sperm from male rats that belonged to the same strain. The CRISPR/Cas9 complex was introduced into the IVF embryos as described above. The gRNA targets were rat *Rosa26*, 5′-GAGTCTTTCTGGAAGATAGG-3′ and rat *Tyr*, 5′-TTTCCAGGATTATGTAATAG-3′. After electroporation, the oocytes were cultured in HTF medium containing 1 × 10^4^ IU/mL, 1 × 10^5^ IU/mL, and 1 × 10^6^ IU/mL of the recombinant ssAAV vector and incubated for 22 h. After co-incubation with ssAAV, one- or two-cell embryos were cultured using mR1ECM medium (ARK Resource) to obtain preimplantation embryos or transferred to oviducts of pseudo-pregnant recipient females. The number of offspring born naturally or by caesarean section was counted after 21 days of gestation.

### Knock in using CRISPR/Cas9 electroporation with large double strand DNA (ldsDNA)

Linearized ldsDNA donor (3.85 kb) was prepared by *Mlu*I-*Sal*I restriction of the plasmid DNA, pAAV_*rRosa26*_CAG_EGFP and purified with kit NucleoSpin Gel and PCR clean-up (Macherey-Nagel, Düren, Germany). The CRISPR/Cas9 complex was introduced into the IVF embryos as described above with 100 ng/μL of purified ldsDNA donor. The gRNA target of rat *Rosa26* was used as above.

### Genotyping of KI preimplantation embryos

Putative KI embryos prepared by ldsDNA electroporation or AAV infection were cultured for 5 days in mR1ECM to develop them into 8-cell embryos to blastocysts. The zona pellucida was removed using acidic Tyrode’s solution (Sigma-Aldrich), and then directly subjected to genomic PCR using Tks Gflex DNA polymerase and the primers listed in Table S1. PCR fragments were electrophoresed and sequenced for further confirmation. For PCR-RFLP analysis, *Tyr* alleles were amplified by PCR and digested using the restriction enzyme *Sna*BI (New England Biolabs), and electrophoresed to detect the KI allele.

### Genotyping of KO/KI offspring

PCR-based genotyping of offspring was performed using a crude lysate of the tail tips, where the genomic DNA was extracted by purification in phenol–chloroform and ethanol precipitation. Approximately 500–1,300 bp genomic fragments containing the on-target site or off-target site were amplified by PCR using Tks Gflex DNA Polymerase and the primers listed in Table S1. For PCR-RFLP analysis, *Tyr* alleles were amplified by PCR and digested using *Sna*BI and electrophoresed to detect the KI allele. Potential off-target sites in the rat genome (RGSC6.0/rn6) were identified using the latest version of the CRISPR Design Tool website (GGGenome: http://gggenome.dbcls.jp/en/rn6/2/). Potential sites that had less than 2 mismatches and/or indel in 20 mer plus PAM sequence of the gRNA were amplified by PCR and sequenced to identify off-target sites in the founders. In addition, a candidate off-target site of the gRNA for disrupting *Tyr* of BN rat (W-off-target: Table S1) was examined as reported previously^31^.

### Statistical analysis

Mean values were compared using one-way analysis of variance. If appropriate, significant differences between means were analysed with Fisher’s exact probability test, where *p* < 0.05 was considered to indicate a significant difference.

## Supplementary information

Supplementary tables and figures
