# Author Correction: Efficient derivation of knock-out and knock-in rats using embryos obtained by *in vitro* fertilization

**DOI:** 10.1038/s41598-020-58515-4

**Published:** 2020-01-30

**Authors:** Arata Honda, Ryoma Tachibana, Kazuya Hamada, Kohtaro Morita, Naoaki Mizuno, Kento Morita, Masahide Asano

**Affiliations:** 1 Institute of Laboratory Animals, Kyoto University Graduate School of Medicine, Yoshidakonoe-cho, Sakyo-ku, Kyoto 606-8501 Japan; 2 RIKEN BioResource Research Center, Tsukuba, Ibaraki 305-0074 Japan; 3 Division of Stem Cell Therapy, Institute of Medical Science, University of Tokyo, Minato-ku, Tokyo 108-8639 Japan

Correction to: *Scientific Reports* 10.1038/s41598-019-47964-1, published online 09 August 2019

This Article contains errors in Table 5. The ‘No. of KI offspring (%)’ value is missing and the ‘No. of offspring with partial insertion (%)’ value should read “1 (5.3)” and not “9 (47.4)”. The correct Table 5 appears below as Table 1.

**Table 1:** Knock-in efficiency of rat offspring after zygote genome editing using electroporation followed by AAV transduction.

Target gene	No. of embryos electroporated	No. of embryos cultured with AAV	No. of embryos survived (%)	No. of embryos transferred	No. of offspring (%)	No. of KO offspring without GFP signal (%)	No. of offspring with GFP signal (%)	No. of KI offspring (%)	No. of offspring with partial insertion (%)	No. of offspring with random integration (%)
*Rosa 26*	376	366	349 (95.4)	180	19 (10.6)	7 (36.8)	12 (63.2)	9 (47.4)	1 (5.3)	2 (10.5)

