# Supplementary material for: Efficient derivation of knock-out and knock-in rats using embryos obtained by in vitro fertilization
Source: Sci Rep. 2019 Aug 9;9:11571. doi: 10.1038/s41598-019-47964-1 (PMC6689013; doi:10.1038/s41598-019-47964-1)
Supplement: Supplementary file 1 — Supplementary tables and figures [file 41598_2019_47964_MOESM1_ESM.pdf]

Supplementary Info.

Efficient derivation of knock-out and knock-in rats using embryos obtained by *in vitro* fertilization

Arata Honda[1,2*], Ryoma Tachibana[1], Kazuya Hamada[1], Kohtaro Morita[1], Naoaki Mizuno[3], Kento Morita[1], Masahide Asano[1]

[1]Institute of Laboratory Animals, Kyoto University Graduate School of Medicine, Yoshidakonoe-cho, Sakyo-ku, Kyoto, 606-8501, Japan.

[2] RIKEN BioResource Research Center, Tsukuba, Ibaraki 305-0074, Japan.

[3]Division of Stem Cell Therapy, Institute of Medical Science, University of Tokyo, Minato-ku, Tokyo 108-8639, Japan

E-mail: honda.arata.7a@kyoto-u.ac.jp

Table S1. gRNAs and PCR primers in this study.

**gRNAs**	Sequence (5' to 3') (Locus)
Tyr -wild-crRNA	TTTCCAGGATTACGTAATAGTGG (chr1:+151097594)
W-off-target	TTTCCAGGAAAAGGTAATAGTGG (chr14:+3768330)
Tyr -repair-crRNA	TTTCCAGGATTATGTAATAGTGG (chr1:+151097594)
Repair-off-target	TTTCCAGGA-TATGTAATGGTGG (chr7:+93476924)
Rosa 26 -crRNA	GAGTCTTTCTGGAAGATAGGCGG (chr4:-145016234)

**Primers**	Forward Primer (5' to 3')	Reverse Primer (5' to 3')	Mutation
Tyr -wild-crRNA	GCTCAAGGTTTAGTTGGGTACT	CTGGCTAGGTTTACTATCTCCTTG	46/46
W-off-target	ATCAGCACTAGACTGGTCAA	GAGAGTCTGTCACAAGCTTC	0/16
Tyr -repair-crRNA	GCATGCAAACAAGTGTGTGTT	GCATTGCAAAACTCACAAAA	3/3
Repair-off-target	CAGGAACCTCAGTCCTGCTC	TCACGTCAGCAATGGTGATT	0/3
Rosa 26 -crRNA	CTGAGGAACGTGCTGAACAA	ACTGTAGCAAGGATCGCAAGTG	19/19

**Primers**	Forward Primer (5' to 3')	Reverse Primer (5' to 3')
Genotyping of GFP-KI into *Rosa26*		
Primer F1-R1	CAGAGAGCCTCGGCTAGGTA	GGGCGTACTTGGCATATGAT
Primer F2-R2	CACTACCTGAGCACCCAGTC	GCAGCTATGGCTCCTCTGTC
Primer F3-R3	CTGGTCGAGCTGGACGGCGACG	CACGAACTCCAGCAGGACCATG
Primer F4-R4	CTGAGGAACGTGCTGAACAA	ACTGTAGCAAGGATCGCAAGTG
Construction of pAAV_*rTyr* -repair		
Wistar *Tyr* amplification	TAAGGCATGGTCTCTGTCTGACT	GTGAGCATGTGCTGGATTAAGGA
5'-homology arm	gacggccagtgaattcGCTAGCtaaggcatggtctctgtctg	GttacgtaaCaAAggtccctcaggtgttcc
3'-homology arm	ccTTtGttacgtaaCccCggTaaccatgacaaagccaaaa	tgattacgccaagcttACGCgtgagcatgtgctggattaa

Mismatches are shown in red. PAM sequences are marked in blue.

For the construction of pAAV_*rTyr* -repair, in-frame silent mutations (upper case) were introduced to the primers.

Table S2. Effect on knock-out efficiency of rat offspring using electroporation at the various times after insemination.

EP time after insemination	No. of embryos electroporated	No. of embryos transferred	No. of offspring (%)	No. of offspring with albino (%)
8-10 h*	224	171	49 (28.7)	49 (100.0)
10 h	65	60	16 (26.7)	16 (100.0)
14 h	70	60	12 (20.0)	12 (100.0)
18 h	65	60	12 (20.0)	12 (100.0)
total	424	351	89 (25.3)	89 (100.0)

* results indicated in Table 4

Table S3. Knock-in efficiency of rat preimplantation embryos after zygote genome editing using ldsDNA or various concentrations of AAV6 (AAV6_*rRosa26*_CAG_EGFP).

Operations	No. of embryos electroporated	No. of embryos survived (%)	No. of embryos developed into morula or blastocyst (%)	No. of embryos with GFP signal (%)	No. of KI embryos /PCR examined (%)
Mock	34	32 (94.1)	7 (21.9)	0 (0.0)	0/2 (0.0)
Rosa 26-KI (ldsDNA)	45	40 (88.9)	9 (22.5)	0 (0.0)	0/8 (0.0)
Rosa 26-KI (ssAAV 10^4 IU/ml)	41	41(100.0)	9 (22.0)	0 (0.0)	0/8 (0.0)
Rosa 26-KI (ssAAV 10^5 IU/ml)	41	40 (97.6)	8 (20.0)	5 (62.5)	2/8 (25.0)
Rosa 26-KI (ssAAV 10^6 IU/ml)	42	40 (95.2)	7 (17.5)	6 (85.7)	5/7 (71.4)

Table S4. Knock-in efficiency of rat preimplantation embryos after zygote genome editing using AAV6 (AAV6_*rTyr*-repair).

Operations	No. of embryos electroporated	No. of embryos survived (%)	No. of embryos developed into 8-cell to blastocyst (%)	No. of KI embryos (%)
Mock	17	17 (100.0)	7 (41.0)	n.d.
Tyr-KI (ssAAV 10^5 IU/ml)	39	38 (94.1)	16 (42.1)	0 (0.0)
Tyr-KI (ssAAV 10^6 IU/ml)	39	37 (95.2)	16 (43.2)	4 (25.0)

Table S5. Knock-in efficiency of rat offspring after zygote genome editing using electroporation followed by AAV transfection.

Operations	No. of embryos electroporated	No. of embryos cultured with AAV (%)	No. of embryos survived (%)	No. of embryos transferred (%)	No. of offspring (%)	No. of offspring with indel mutation (%)	No. of KI offspring (%)
Tyr-KI (ssAAV 10^6IU/ml)	42	42 (100.0)	42 (100.0)	42 (100.0)	3 (7.1)	3 (100)	1 (33.3)

		PAM gRNA
WT		ggaccactattacgtaatcctggaaacca
♂10		n.d.
♂11	Allele 1	ggaccactaattacgtaatcctggaaacca
	Allele 2	ggacca------Δ54------tcagca
♂12	Allele 1,2	ggaccactatttttacgtaatcctggaaacca
♂13	Allele 1,2	ggaccactaaattacgtaatcctggaaacca
♂14		n.a.
♂15	Allele 1	ggaccactatttacgtaatcctggaaacca
	Allele 2	gga------Δ41------caaagccaa
♂16	Allele 1,2	ggaccactatttttacgtaatcctggaaacca
♂17	Allele 1,2	ggaccactatattacgtaatcctggaaacca
♂18	Allele 1,2	ggaccactaattacgtaatcctggaaacca
♂19	Allele 1	ggaccac---tacgtaatcctggaaacca
	Allele 2	ggacca------Δ23------tgacaa
♂20	Allele 1,2	ggacca------Δ28------agcca
♂21	Allele 1,2	ggaccactatattacgtaatcctggaaacca
♂22	Allele 1,2	gg--Δ30--tgg------t-Δ13--ccc
♂24	Allele 1	ggaccactatttacgtaatcctggaaacca
	Allele 2	ggaccactaaattacgtaatcctggaaacca
♂25	Allele 1,2	ggaccactatttacgtaatcctggaaacca
♂26	Allele 1	ggaccacta---cgtaatcctggaaacca
	Allele 2	ggaccactagttacgtaatcctggaaacca
♂27		n.d.
♂28		n.d.

		PAM gRNA
WT		ggaccactattacgtaatcctggaaacca
♀8	Allele 1	ggaccactatattacgtaatcctggaaacca
	Allele 2	cat-Δ113-aca-Δ16-tacgtaatcctggaaacca
♀9	Allele 1	ggaccactaattacgtaatcctggaaacca
	Allele 2	ccctt--Δ117--gtaatcctggaaacca
♀10	Allele 1	ggaccactaattacgtaatcctggaaacca
	Allele 2	ggaccactatattacgtaatcctggaaacca
♀11	Allele 1,2	ggaccac-----Δ41-----aggctcc
♀12	Allele 1,2	ggaccactaatttacgtaatcctggaaacca
♀13	Allele 1,2	ggaccac------gtaatcctggaaacca
♀14		n.d.
♀15	Allele 1	ggaccgtgatatgcccgcctgagggacca
	Allele 2	ggacca-----Δ121-----atttcagc
♀16	Allele 1,2	acacctg-----Δ24-----gaaacca
♀17		n.a.
♀18	Allele 1	ggaccactat-acgtaatcctggaaacca
	Allele 2	acctg--Δ15--gtaatcctggaaacca
♀19	Allele 1	caca--Δ81--tcctggaaaccatggac
	Allele 2	cgcattttgcataaa-Δ12-gatcatttgca gcaagatcagaaagtcataatagccatcag gttttatgtgatggaa--Δ37--cccctgag gtccctcagga--Δ44--ccagtatgaat
♀20	Allele 1	ggaccactagttacgtaatcctggaaacca
	Allele 2	tca--t----tgtgat--Δ34--gaaacca
♀21	Allele 1,2	ggaccactatcctacgtaatcctggaaacca

Figure S1. Sequence analysis of PCR products amplified from albino offspring genomic DNA had a wide variety of indel mutations at the targeted *Tyr* exon2. The magnified view illustrates the gRNA binding sites (blue) and PAM sequences (red). All of the sequenced offspring had biallelic indel mutations (green). Insertions are underlined. n.d.: not determined by sequencing analysis due to complex mutations. n.a.: not amplified due to problems with PCR.

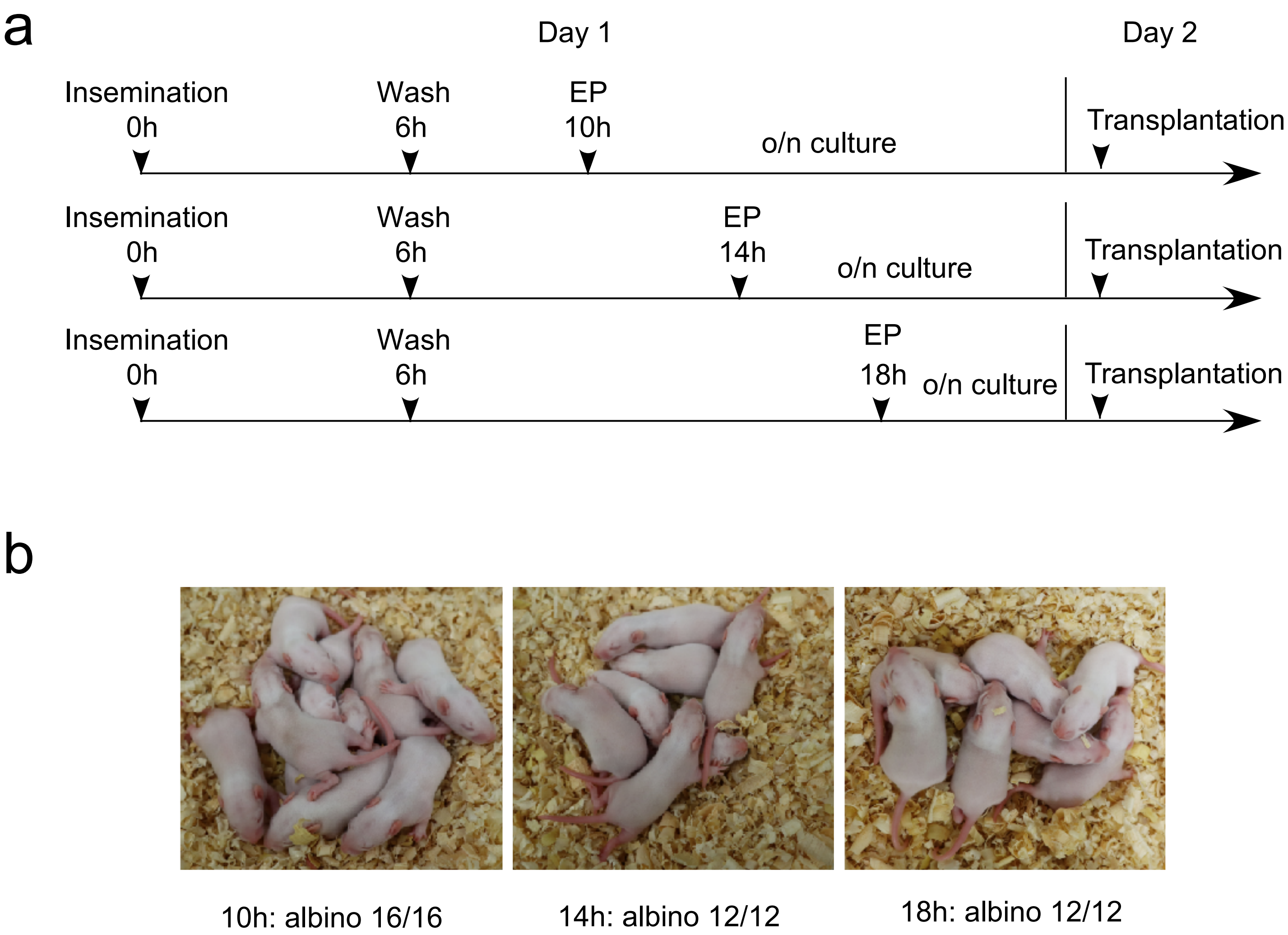

Figure S2. (**a**) Time scale representation of CRISPR/Cas9 introduction by electroporation (EP) at the various times 10, 14, and 18 h after insemination. (**b**) All pups derived were of the albino phenotype.

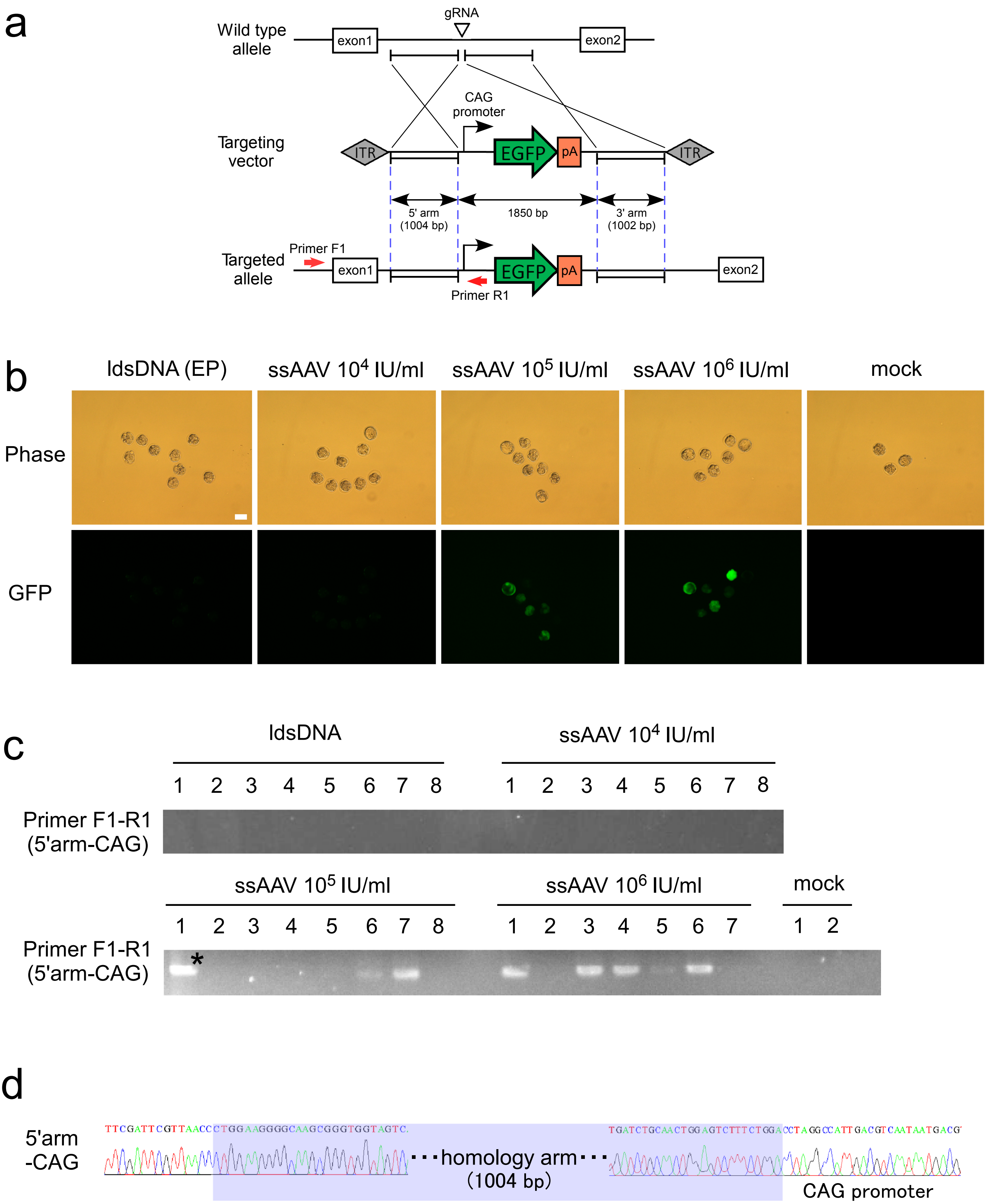
a
Wild type allele
gRNA
exon1
exon2
CAG promoter
Targeting vector
ITR
EGFP
pA
5' arm (1004 bp)
1850 bp
3' arm (1002 bp)
Primer F1
Targeted allele
Primer R1
b
ldsDNA (EP)
ssAAV 10^4 IU/ml
ssAAV 10^5 IU/ml
ssAAV 10^6 IU/ml
mock
Phase
GFP
c
ldsDNA
ssAAV 10^4 IU/ml
1 2 3 4 5 6 7 8
Primer F1-R1 (5'arm-CAG)
ssAAV 10^5 IU/ml
ssAAV 10^6 IU/ml
mock
*
d
5'arm -CAG
homology arm (1004 bp)
CAG promoter

Figure S3. (**a**) Schematic representation of the targeting strategy. The donor ssAAV vector containing the 1,850-bp CAG-EGFP cassette was flanked by approximately 1,000-bp homology arms next to the gRNA target. Arrows indicate the primers used for genomic analysis. (**b**) Transfection of donor DNA by the electroporation of long double-strand (lds) DNA and co-cultured with AAV6_*rRosa26*_CAG_EGFP at the various (1 x 10^4 IU/mL, 1 x 10^5 IU/mL, and 1 x 10^6 IU/mL) concentrations. Compared with that of the mock-treated embryos, 1 x 10^5 IU/mL and 1 x 10^6 IU/mL showed high EGFP expression. (**c**) Representative genotyping of the embryos showed the existence of the KI allele at 1 x 10^5 IU/mL (2/8) and 1 x 10^6 IU/mL (5/7). *Band emerged ssAAV 10^5 IU/mL-1 was not the KI allele. (d) Representative of Sanger sequencing of the precise insertion of knock in cassettes in *Rosa26*-CAG-EGFP knock in embryos (ssAAV 10^5 IU/mL-7). Homology direct repair was confirmed by Sanger sequencing of 5' junctional regions. Blue box, homology arm.

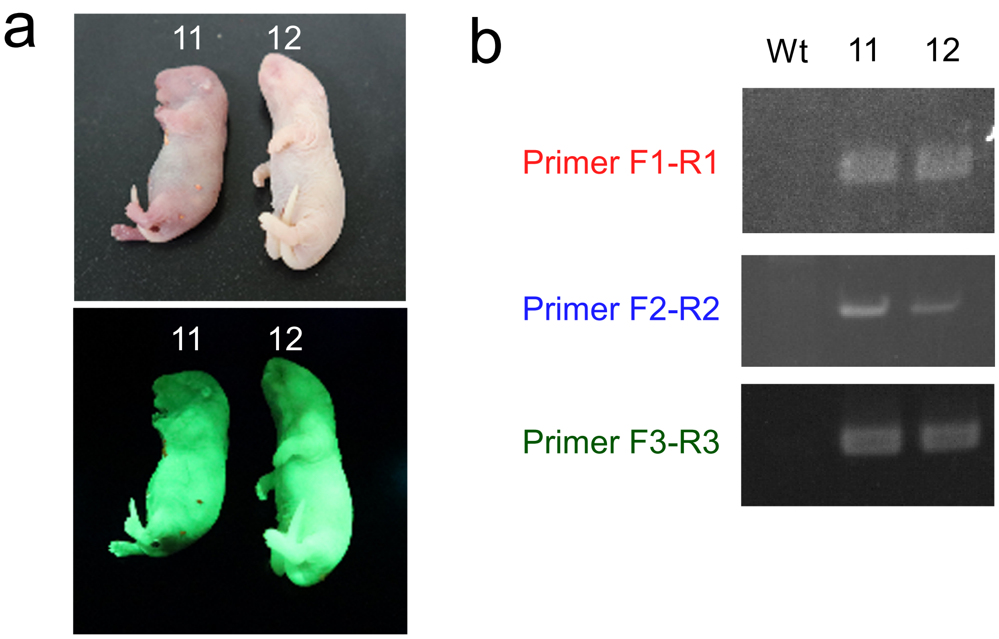

Figure S4. (**a**) Two stillborn pups had uniform GFP signals over their whole bodies. (**b**) Representative genotyping of stillborn pups. Primers flanking the 5'- (Primer F1-R1) and 3'- (Primer F2-R2) junctional regions, and GFP cassette (Primer F3-R3) were amplified. These primers targeted the allele at a precise locus.

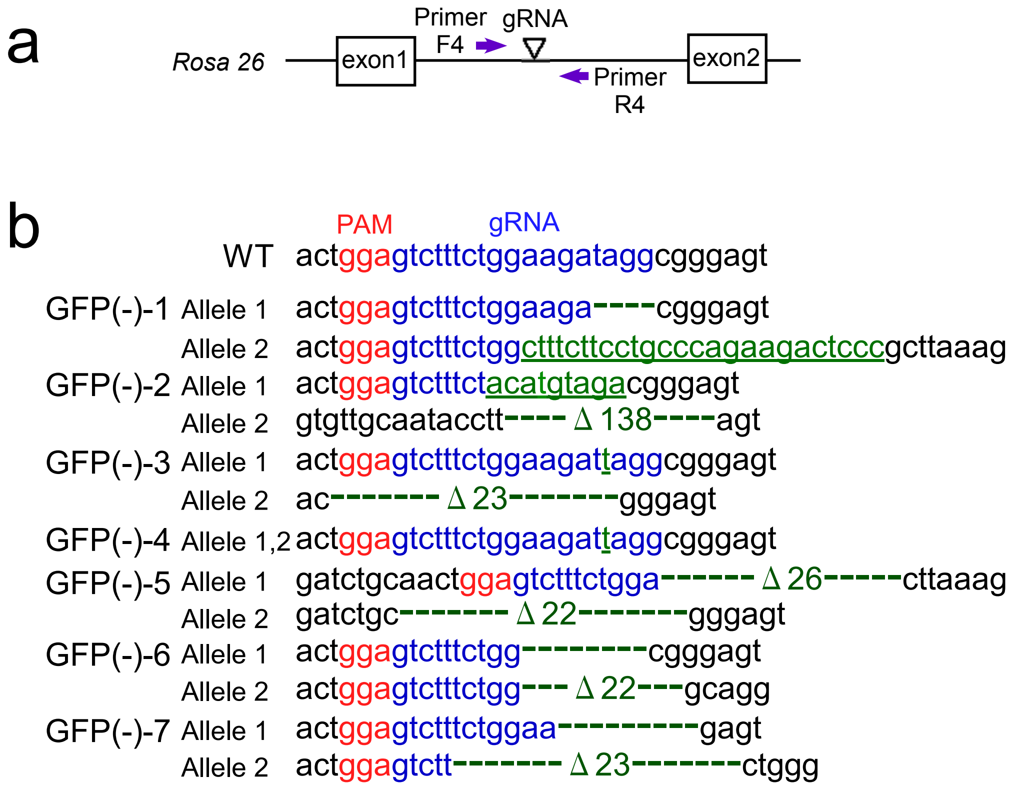

Figure S5. (**a**) Schematic representation of the *Rosa26* target site. Arrows indicate the primers for PCR amplification and sequencing. (**b**) Sequence analysis of PCR products amplified from the GFP (-) offspring genomic DNA had a wide variety of indel mutations at the targeted *Rosa26* site. The magnified view illustrates the gRNA binding sites (blue) and the PAM sequences (red). All of the sequenced offspring had biallelic indel mutations (green). Insertions are underlined.

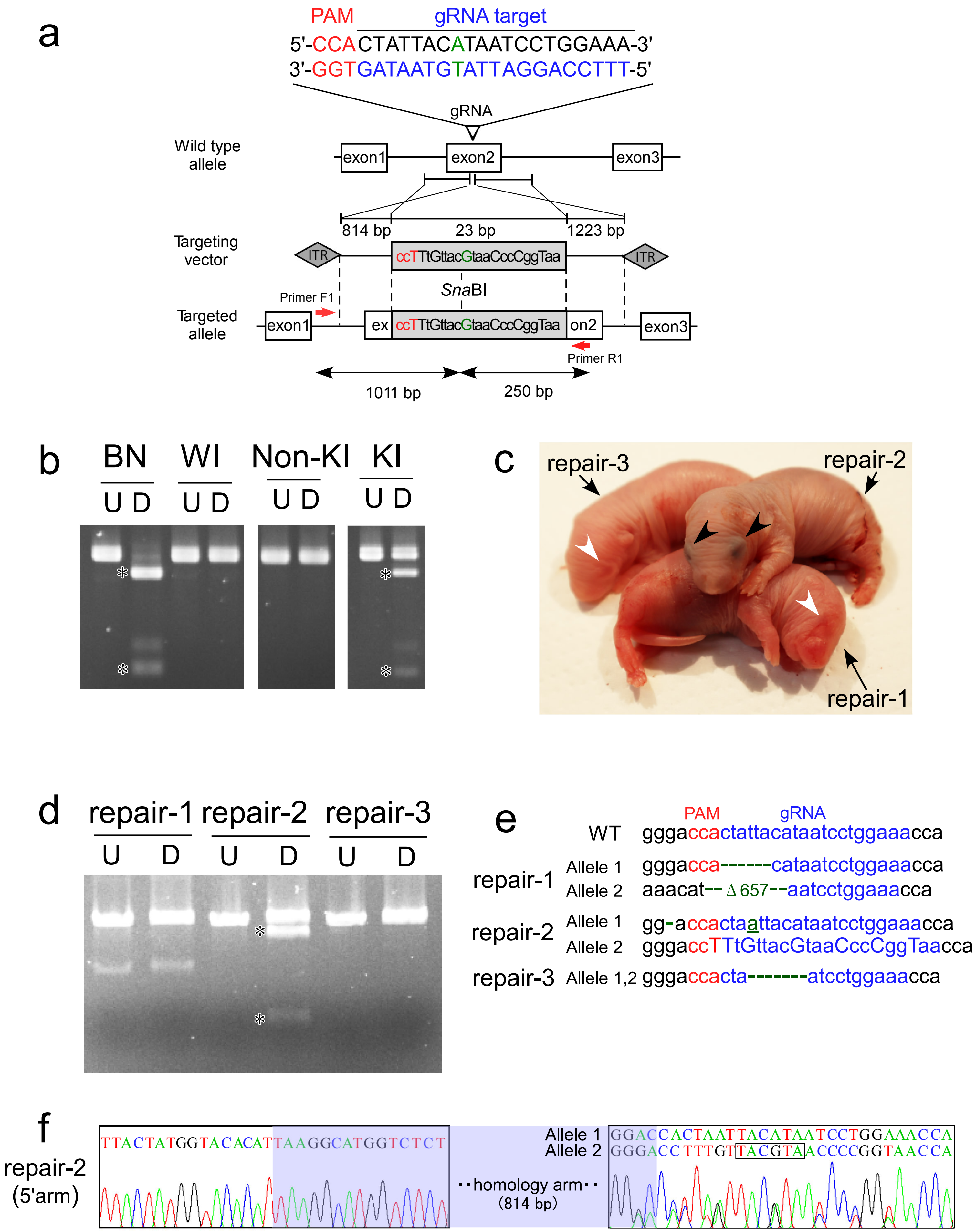

a
PAM
gRNA target
5'-CCACTATTACATAATCCTGGAAA-3'
3'-GGTGATAATGTATTAGGACCTTT-5'
gRNA
Wild type allele
exon1
exon2
exon3
814 bp
23 bp
1223 bp
Targeting vector
ITR
ccTTtGttacGtaaCccCggTaa
ITR
SnaBI
Primer F1
Targeted allele
exon1
ex
ccTTtGttacGtaaCccCggTaa
on2
exon3
Primer R1
1011 bp
250 bp
b
BN
WI
Non-KI
KI
U D
U D
U D
U D
c
repair-3
repair-2
repair-1
d
repair-1
repair-2
repair-3
U D
U D
U D
e
PAM
gRNA
WT gggaccactattacataatcctggaaacca
repair-1 Allele 1 gggacca------cataatcctggaaacca
Allele 2 aaacat--Δ657--aatcctggaaacca
repair-2 Allele 1 gg-accactaattacataatcctggaaacca
Allele 2 gggaccTTtGttacGtaaCccCggTaacca
repair-3 Allele 1,2 gggaccacta-------atcctggaaacca
f
repair-2 (5'arm)
TTACTATGGTACACATTAAGGCATGGTCTCT
Allele 1 GGACCACTAATTACATAATCCTGGAAACCA
Allele 2 GGGACCTTTGTTACGTAACCCCGGTAACCA
··homology arm··
(814 bp)

Figure S6. (**a**) Schematic representation of the targeting strategy. The donor ssAAV vector (AAV6_*Tyr*-repair) containing the homology arms includes the *Tyr*_repair cassette that has the corrected sequences containing the in-frame silent mutation to provide an *Sna*BI site for RFLP analysis. The target sequence of *Tyr* exon 2 recognized by gRNA is overlined and the PAM sequence is marked in red. The target nucleotide ‘A’ marked in green is a key nucleotide for tyrosinase activity, and nucleotide replacement at this position leads to the pigmented phenotype. Silent mutations introduced to provide the *Sna*BI site and to prevent recognizing crRNA of KI allele are represented in uppercase. Arrows indicate the primers used for genomic analysis. (**b**) Representative of RFLP analysis for preimplantation embryos generated by the introduction of donor ssAAV6 (AAV6_*Tyr*-repair). The 1261-bp amplicon of *Tyr* flanking homology arms and intron 1 were digested with *Sna*BI. Amplicon derived from *Tyr*-repaired allele resulted in 1011-bp and 250-bp products. Genomic DNA from BN and Crlj:WI (WI) were used as controls. Embryos of BN strain and KI (KI) produced 1011-bp and 250-bp products (asterisks) by *Sna*BI digestion. U: undigested, D: digested. (**c**) Offspring derived by the transfection of AAV6_*Tyr*-repair. Offspring having repair-2 with pigmented eyes (closed arrowheads). By contrast, repair-1 and repair-3 have non-pigmented eyes (open arrowheads). (**d**) Representative of RFLP analysis for the offspring repair-1, repair-2, and repair-3. The amplicon derived from repair-2 produced *Tyr*-repaired allele by the *Sna*BI digestion (1011-bp and 250-bp products: asterisks). (**e**) Sequence analysis of PCR products amplified from the offspring (repair-1, repair-2, and repair-3) genomic DNA had a wide variety of indel mutations at the targeted *Tyr* site. Repair-2 has a KI allele (Allele 2). In-frame silent mutations are represented in uppercase. The magnified view illustrates the gRNA binding sites (blue) and the PAM sequences (red). All of the sequenced offspring had indel mutations (green). Insertions are underlined. (**f**) Representative of Sanger sequencing of the precise insertion of knock in cassettes in an offspring, repair-2. Homology direct repair was confirmed by Sanger sequencing of 5' junctional regions. Blue box, homology arm. Boxed sequence, *Sna*BI site emerged in the allele 2.

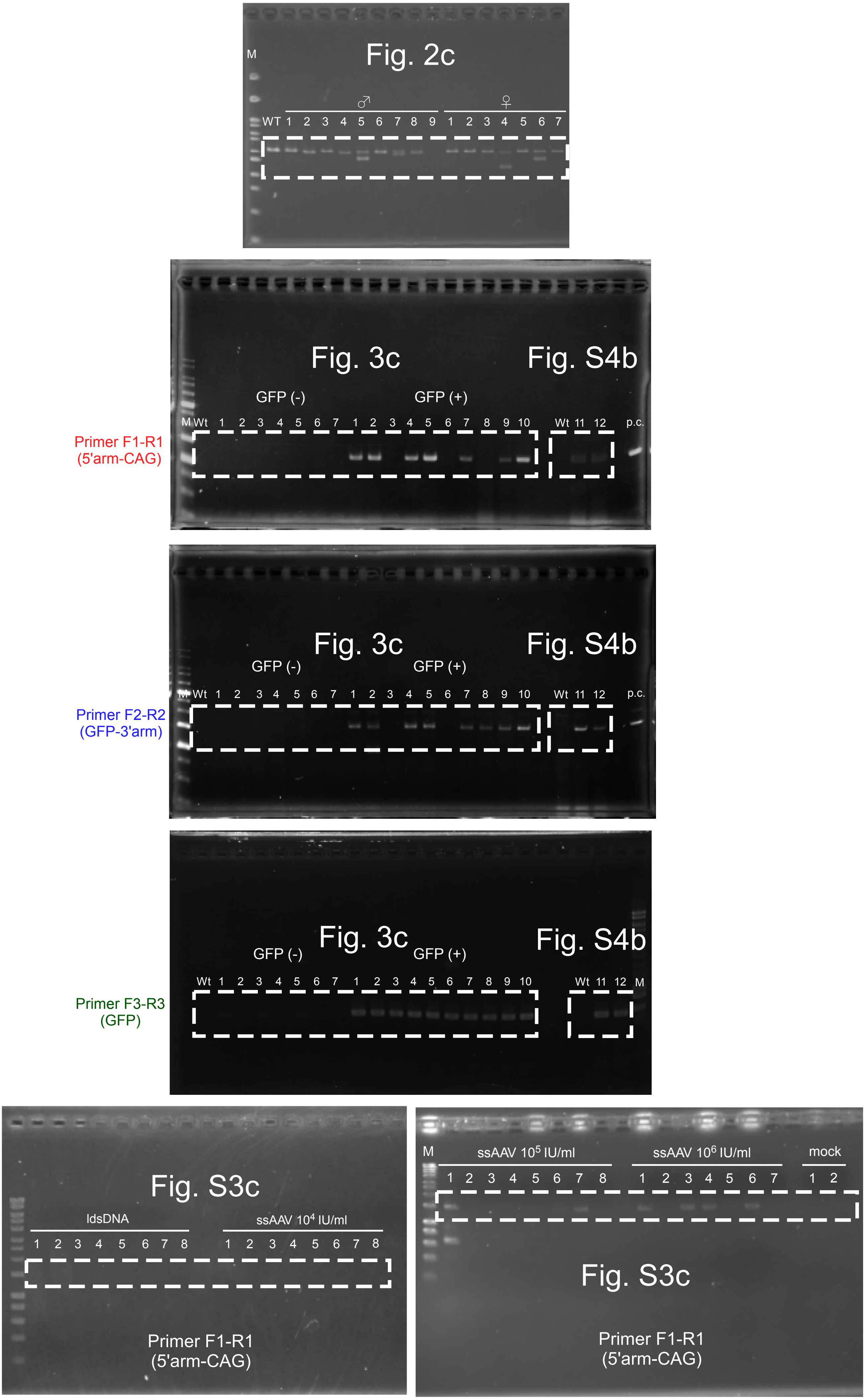

Figure S7. Full-length agarose gels for Figs. 2c, 3c, S3c, and S4b.

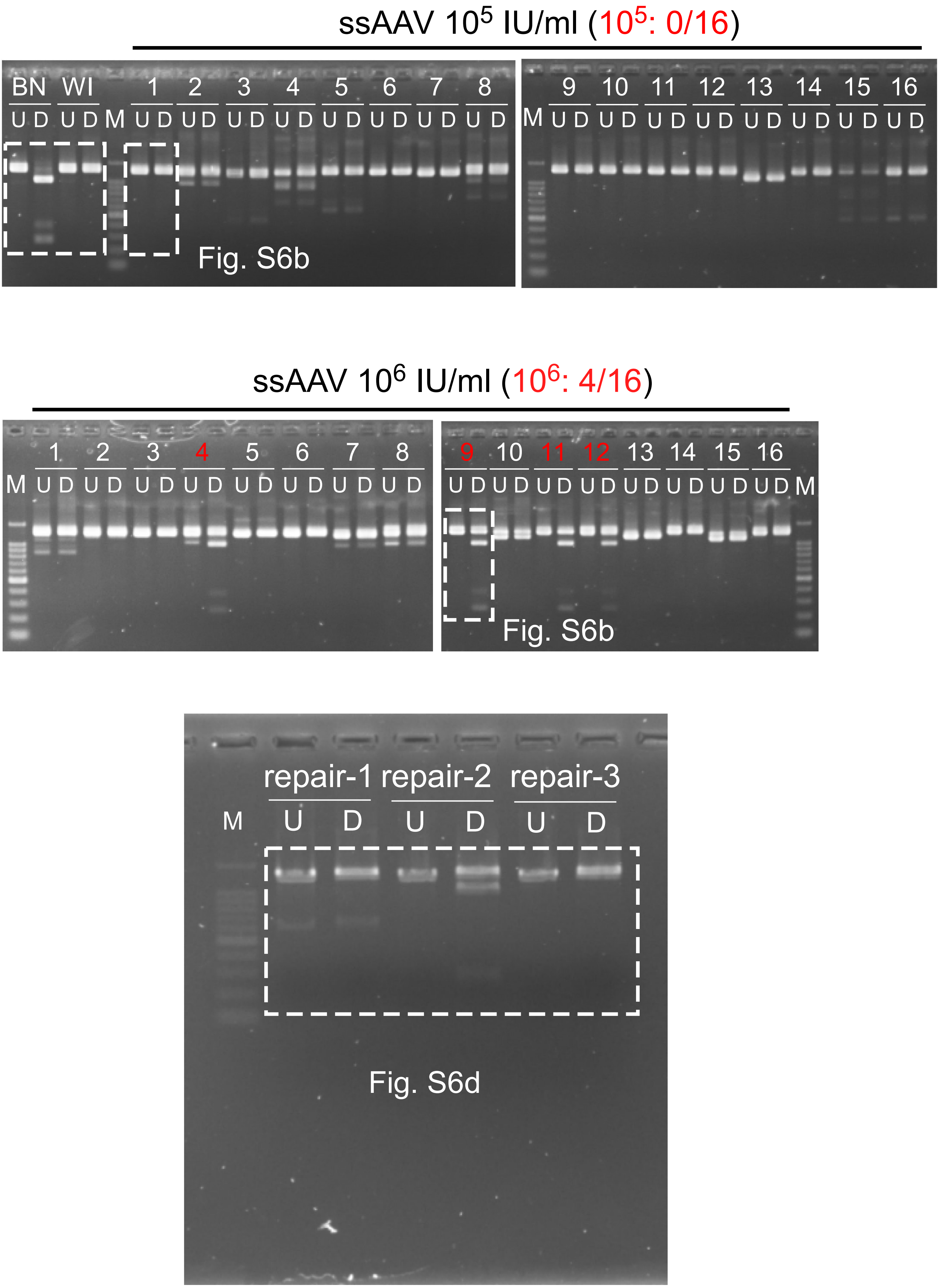

Figure S8. Full-length agarose gels for Fig. S6b and S6d.
